# Supplementary material for: The Effect of Ametropia on Glaucomatous Visual Field Loss
Source: J Clin Med. 2021 Jun 25;10(13):2796. doi: 10.3390/jcm10132796 (PMC8268842; doi:10.3390/jcm10132796)
Supplement: Supplementary file 1 [file jcm-10-02796-s001.zip › jcm-1263686-supplementary.pdf]

SE coefficients

			−0.104	−0.075	−0.054	−0.089		
		−0.053	−0.014	−0.005	0.002	0.004	−0.037	
	−0.067	−0.012	−0.002	0.021	0.026	0.028	0.012	−0.038
−0.109	−0.027	−0.002	0.027	0.03	0.03	0.035		0.059
−0.128	−0.048	−0.02	−0.003	0.005	0.019	0.025		0.163
	−0.066	−0.01	−0.001	0.01	0.028	0.026	0.067	0.033
		−0.031	−0.002	0.003	0.019	0.042	0.022	
			−0.054	−0.032	−0.028	−0.035		

MD coefficients

			0.531	0.502	0.488	0.471		
		0.571	0.503	0.515	0.516	0.445	0.421	
	0.638	0.563	0.548	0.56	0.546	0.48	0.383	0.295
0.635	0.652	0.636	0.598	0.516	0.335	0.422		0.226
0.576	0.586	0.555	0.471	0.275	0.122	0.165		0.22
	0.564	0.504	0.488	0.444	0.402	0.374	0.299	0.279
		0.506	0.456	0.461	0.421	0.344	0.355	
			0.477	0.431	0.403	0.395		

Interaction coefficients

			0.004	0.005	0.007	0.008		
		0	−0.001	0	0.003	0.005	0.008	
	−0.001	−0.004	−0.007	−0.007	−0.002	0.001	0.001	0.011
−0.003	−0.003	−0.008	−0.009	−0.007	−0.002	−0.001		0.008
−0.002	−0.003	−0.009	−0.01	−0.007	−0.005	−0.001		0.008
	−0.004	−0.007	−0.007	−0.006	−0.002	−0.002	0.002	0.011
		−0.002	−0.002	−0.001	0.002	0.006	0.012	
			0.004	0.005	0.008	0.011		

Supplemental Figure S1: Regression coefficients for each of the 52 visual field locations.

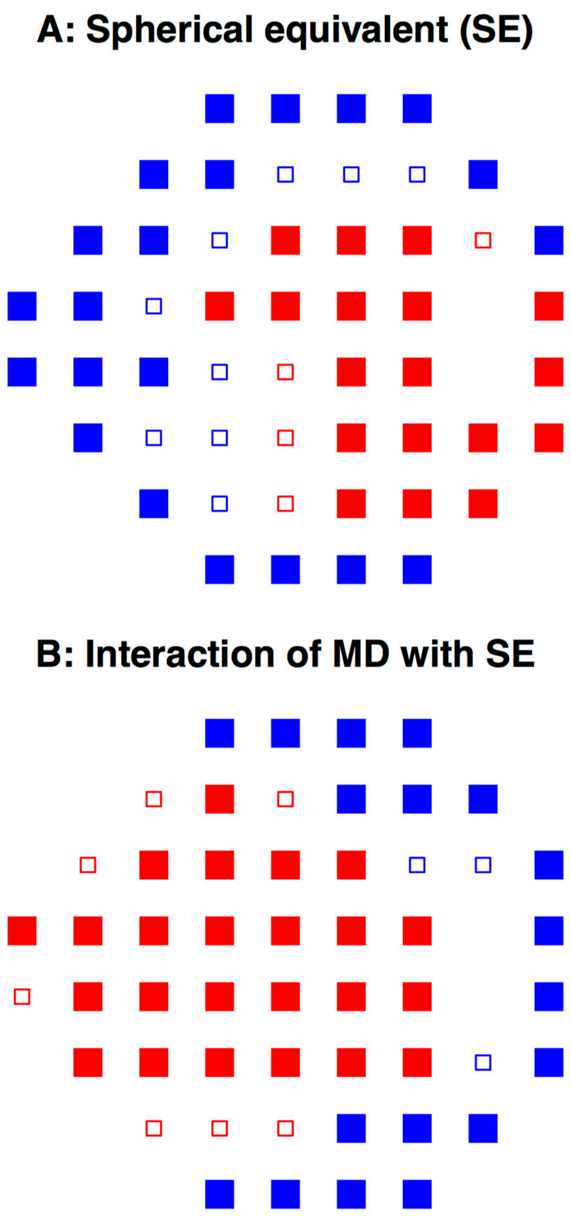

Supplemental Figure S2: Impact of spherical equivalent (SE) on visual field pattern deviations that is not explained by glaucoma severity (mean deviation, MD) (**A**) and interaction effects between glaucoma severity (MD) and SE (**B**) when excluding patients with ages >80 years.

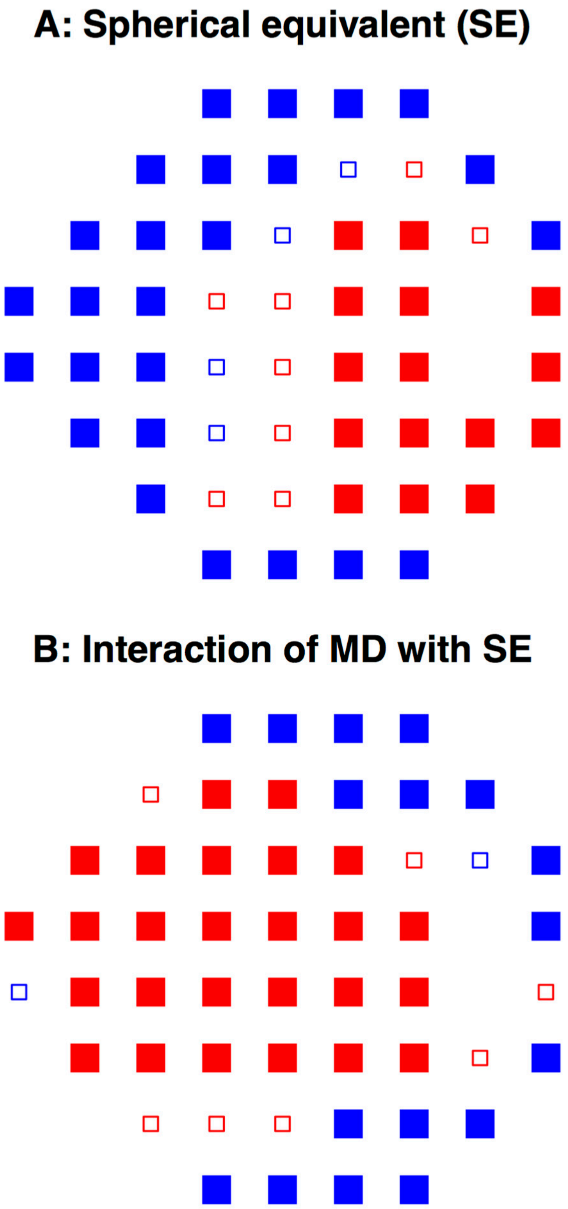

Supplemental Figure S3: Impact of spherical equivalent (SE) on visual field pattern deviations that is not explained by glaucoma severity (mean deviation, MD) (**A**) and interaction effects between glaucoma severity (MD) and SE (**B**) when excluding VFs with MD < −18 dB.

A: Spherical equivalent (SE)

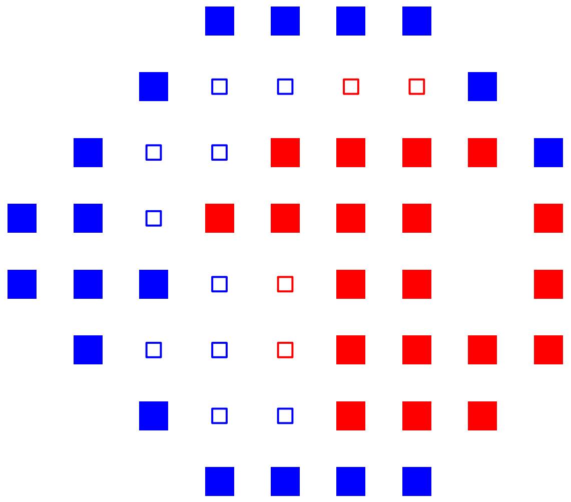

B: Interaction of MD with SE

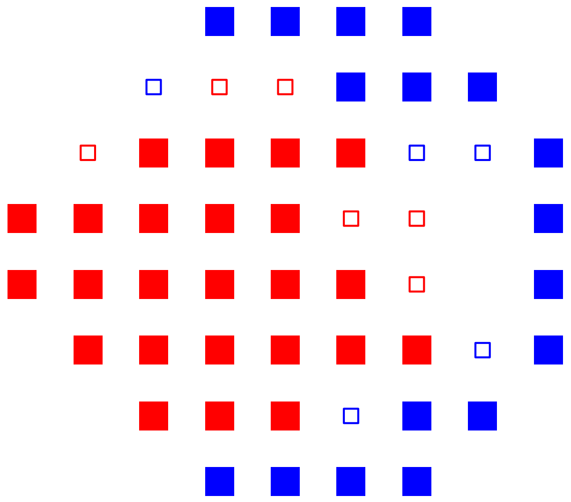

Supplemental Figure S4: Impact of spherical equivalent (SE) on visual field pattern deviations that is not explained by glaucoma severity (mean deviation, MD) (**A**) and interaction effects between glaucoma severity (MD) and SE (**B**) after excluding eyes with lower absolute refractive error (−1.5 D ≤ SE ≤ +1.0 D)
